# Photocatalytic activity of BiFeO_3_/ZnFe_2_O_4_ nanocomposites under visible light irradiation†

**DOI:** 10.1039/c7ra13380d

**Published:** 2018-02-13

**Authors:** B. Safizade, S. M. Masoudpanah, M. Hasheminiasari, A. Ghasemi

**Affiliations:** School of Metallurgy & Materials Engineering, Iran University of Science and Technology (IUST) Tehran Iran masoodpanah@iust.ac.ir +98 21 77240480 +98 21 77240540

## Abstract

Herein, BiFeO_3_/ZnFe_2_O_4_ nanocomposites were synthesized *via* a glyoxylate precursor method using a two-pot approach. Phase evolution is investigated by X-ray diffraction and Raman spectroscopy, which confirm that no impurity phases are formed between BiFeO_3_ and ZnFe_2_O_4_ following calcination at 600 °C. The specific surface area characterized by N_2_ adsorption–desorption isotherms decreases from 30.56 to 13.13 m^2^ g^−1^ with the addition of zinc ferrite. In contrast, the magnetization increases from 0.28 to 1.8 emu g^−1^ with an increase in the amount of ZnFe_2_O_4_. The composites show strong absorption in the visible region with the optical band gap calculated from the Tauc's plot in the range from 2.17 to 2.22 eV, as measured by diffuse reflectance spectroscopy. Furthermore, the maximum efficiency for the photodegradation of methylene blue under visible light is displayed by the composite containing 25 wt% ZnFe_2_O_4_ due to the synergic effect between BiFeO_3_ and ZnFe_2_O_4_, as confirmed by photoluminescence spectroscopy.

## Abstract 2

BiFeO_3_-25 wt% ZnFe_2_O_4_ exhibits a low specific surface area, high magnetization, and maximum photocatalytic efficiency of 97%.

## Graphical abstract

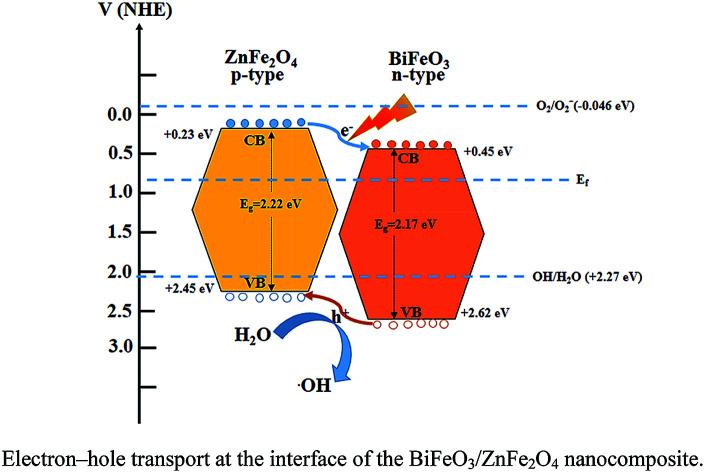

## 1. Introduction

In recent years, scientific research has been focused on new visible light photocatalysts based on semiconductors to address the increasing environmental pollution and energy demands by efficient utilization of solar energy.^1,2^ To date, various metal oxides (ZnO^3^ and TiO_2_ ^4^) and metal sulfides (ZnS^5^) have been studied to efficiently degrade harmful organic pollutants and for hydrogen production through water splitting under UV light irradiation.^4^ However, the UV region spans only 5% of the entire solar spectrum, restricting their applications. As a result of band gap engineering improvement, composites can be fabricated by coupling two narrow band gap semiconductors, which have attracted considerable attention for the development of efficient visible light photocatalysts.^6–8^

Bismuth ferrite (BiFeO_3_), which has potential applications in sensors, actuators, and digital memory, is a well-known multiferroic material simultaneously possessing ferroelectric and ferromagnetic ordering at room temperature.^9,10^ Furthermore, BiFeO_3_ displays a distinct photovoltaic effect with an open circuit voltage of 0.8–0.9 V as a working solar device, which represents a new potential application.^11,12^ Due to its relatively narrow band gap of 2.2 eV, BiFeO_3_ has been considered as a possible visible light photocatalyst under solar light irradiation for the photodegradation of organic contaminants.^13,14^ However, its quantum yield is poor due to the rapid recombination of the photogenerated electron–hole pairs that limits its practical use in photocatalytic applications.^15,16^ Therefore, many strategies have been developed to enhance the photocatalytic efficiency of BiFeO_3_ by modifying the size and morphology of its particles, cation doping, and coupling with other semiconductors.^17–19^ For instance, several semiconductors such as g-C_3_N_4_, carbon nanofiber, graphene, CuO and ZnO have been coupled with BiFeO_3_ to improve its photogenerated electron–hole separation, thus enhancing its interfacial charge transfer the efficiency.^6,20–27^

Spinel magnetic zinc ferrite (ZnFe_2_O_4_) with a narrow band gap of 1.92 eV exhibits a significant photoresponse in the visible light region and has been utilized in gas sensors, catalysts and semiconductor photocatalysts.^1^ Furthermore, the magnetic properties of ZnFe_2_O_4_ can be used to recycle photocatalysts by the application of a magnetic field, making it an interesting product in the industrial photodegradation of organic pollutants.^7,28^ To the best of our knowledge, there are no reports on the synthesis and application of BiFeO_3_/ZnFe_2_O_4_ nanocomposites for pollutant degradation under visible light irradiation. Uniyal and Yadav only reported the dielectric and magnetic properties of BiFeO_3_/ZnFe_2_O_4_ composites synthesized *via* the sol–gel method as a function of annealing temperature.^29^

Herein, we report the structure, microstructure, magnetic properties and photocatalytic performances of BiFeO_3_/ZnFe_2_O_4_ composites synthesized *via* the glyoxylate precursor method. The optimum amount of ZnFe_2_O_4_ is determined to maximize the photocatalytic activity of BiFeO_3_ powder.

## 2. Experimental procedure

Starting materials of Fe(NO_3_)_3_·9H_2_O (>99%), Bi(NO_3_)_2_·5H_2_O (>99%), Zn(NO_3_)_2_·6H_2_O (>99%), 1,2-ethanediol (OH(CH_2_)_2_OH) and nitric acid (HNO_3_, 68 wt%) of analytical grade were provided by Merck & Co.

BiFeO_3_ powder was prepared *via* the glyoxylate precursor method in which the required amount of Fe(NO_3_)_3_·9H_2_O was dissolved in 1,2-ethanediol (ethylene glycol) and then added to 15 mL of 3 mol L^−1^ nitric acid solution containing Bi(NO_3_)_3_·5H_2_O under magnetic stirring at 100 °C. The ethylene glycol : NO_3_^−^ (EG/NO_3_) molar ratio was set to 2.5 : 1. Evolving bubbles of brown nitrogen oxide (NO_*x*_) indicated the initiation of the redox reaction between the NO_3_^−^ anions and OH groups of diol. After drying at 130 °C, the precursor was calcined at 600 °C for 1 h in ambient air.^30^ ZnFe_2_O_4_ powder was produced by dissolving Zn(NO_3_)_2_·6H_2_O and Fe(NO_3_)_3_·9H_2_O in ethylene glycol under magnetic stirring at 100 °C. Once the NO_*x*_ bubbles disappeared, the solution precursor was dried at 130 °C and then calcined at 600 °C for 1 h in air. BiFeO_3_/ZnFe_2_O_4_ composites were synthesized *via* a two-pot approach in which the required amount of previously synthesized BiFeO_3_ powder was added to the solution precursor of zinc ferrite, where the dried precursor was calcined at 600 °C for 1 hour.

Phase evolution was investigated using a PANalytical X'pert X-ray diffractometer (XRD) with monochromatic CuKα radiation. Raman analysis was performed on the powders using a WiTec Alpha 300R instrument (Nd:YAG laser source: *λ* = 532 nm and 0.7 MW power, and range: 100–900 cm^−1^). The morphology and microstructure of the powders were observed using a TESCAN Vega II scanning electron microscope (SEM). The specific surface areas of the as-prepared powders were determined according to the Brunauer–Emmett–Teller (BET) method with nitrogen adsorption at 77 K using a PHS-1020 instrument after degassing at 250 °C for 5 h. The Barrett–Joyner–Halenda (BJH) cumulative pore volume was calculated from the adsorption branch of the isotherms. The equivalent particle size was calculated based on the BET surface area as follows:1
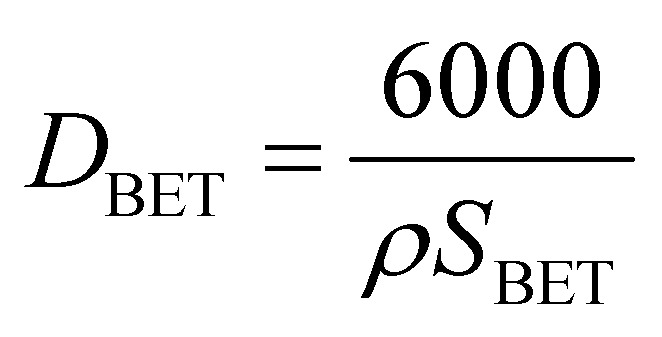
where, *D*_BET_ is the equivalent particle size (nm), *ρ* is the theoretical density and *S*_BET_ stands for the BET surface area (m^2^ g^−1^). A vibrating sample magnetometer (Meghnatis Daghigh Kavir Kashan Co., Iran) with a maximum field of 10 kOe was employed to measure the magnetic properties at room temperature. UV-vis absorption spectra were recorded on a Shimadzu UV-vis-52550 spectrophotometer in the wavelength range of 300−800 nm. Room temperature photoluminescence spectra (PL) were obtained on a fluorescence spectrophotometer (F-4600, Hitachi, Japan) at an excitation wavelength of 210 nm.

The photocatalytic activity of the BiFeO_3_/ZnFe_2_O_4_ nanocomposites was evaluated by the degradation of methylene blue (MB) in aqueous solution under visible light radiation. Two 100 W xenon lamps with a cutoff ultraviolet filter (*λ* = 420 nm) were introduced as the visible light source. In each experiment, 0.1 g of photocatalyst was added to 100 mL of methylene blue solution at a concentration of 15 mg L^−1^. In addition, the solution pH was adjusted to 2 by adding HCl to obtain the maximum MB adsorption on the catalyst surface,^14^ as shown in the ESI.† The suspension was stirred in the dark for 60 min to establish the adsorption/desorption equilibrium, then the solution was irradiated under visible light. At appropriate time intervals, about 5 mL of suspension was sampled, where the solid phase was separated from the solution *via* centrifugation at 4000 rpm for 20 min. The concentration of each degraded solution was monitored on a PG Instruments Ltd T80-UV/vis spectrophotometer.

## 3. Results and discussion

**Fig. 1:**
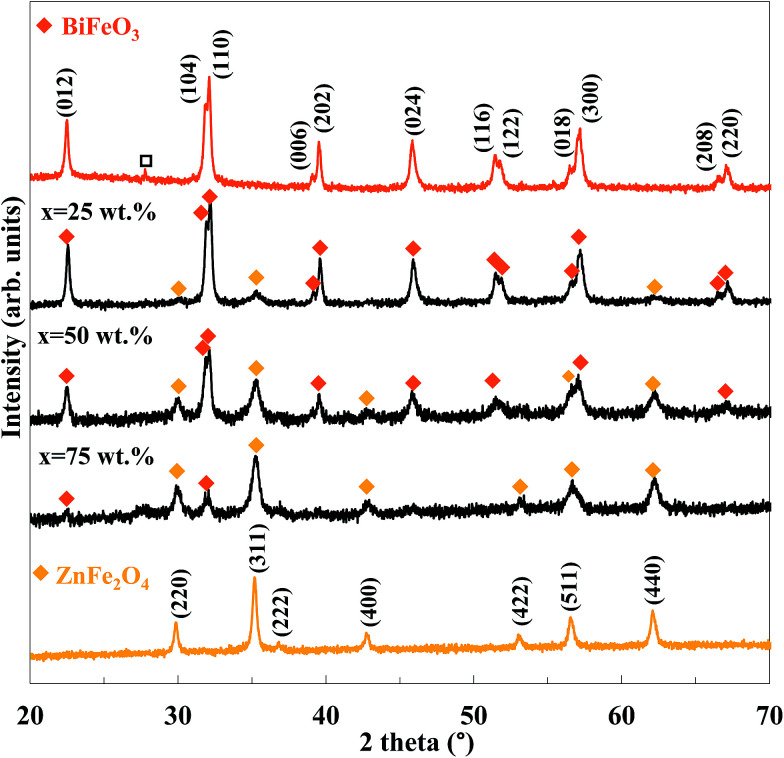
XRD patterns of the BiFeO_3_–*x*ZnFe_2_O_4_ composites as a function of ZnFe_2_O_4_ content (x) (Bi_2_Fe_4_O_9_).

Fig. 1 shows the XRD patterns of the pure BiFeO_3_, pure ZnFe_2_O_4_ and the BiFeO_3_–*x*ZnFe_2_O_4_ composites. The indexed diffraction peaks of ZnFe_2_O_4_ are (220), (311), (400), (422), (511), (440) and (533) which match well with the cubic spinel structure having the *Fd*3̄*m* space group and are in good agreement with the standard JCPDS card no. 22-1012. Pure BiFeO_3_ shows indexed diffraction peaks corresponding to a rhombohedral phase with the *R*3*c* space group (JCPDS no. 86-1518), which indicates well crystallized BiFeO_3_ nanoparticles were produced by the glyoxylate precursor method. However, some impurity Bi_2_Fe_4_O_9_ phases (JCPDS card no. 42-0181) were also observed with BiFeO_3_. The chemical synthesis of BiFeO_3_ typically leads to the formation of impurities, may be due to its chemical kinetics.^31^ After compositing with 25 wt% ZnFe_2_O_4_, a weak diffraction peak at 2*θ* = 35.32° corresponding to the (311) reflection peak of ZnFe_2_O_4_ appeared. With an increase in the zinc ferrite content, the diffraction peaks of ZnFe_2_O_4_ became clearer and stronger, and the impurity peak disappeared. Furthermore, no impurity species were formed between BiFeO_3_ and ZnFe_2_O_4_ during the calcination process, which indicates that ZnFe_2_O_4_ was successfully loaded on the BiFeO_3_ particles without destroying its crystal structure. The amount of BiFeO_3_ and ZnFe_2_O_4_ phases in the composites was calculated by Rietveld refinement, which is in agreement with the nominal values, as typically shown in the ESI.†

**Fig. 2:**
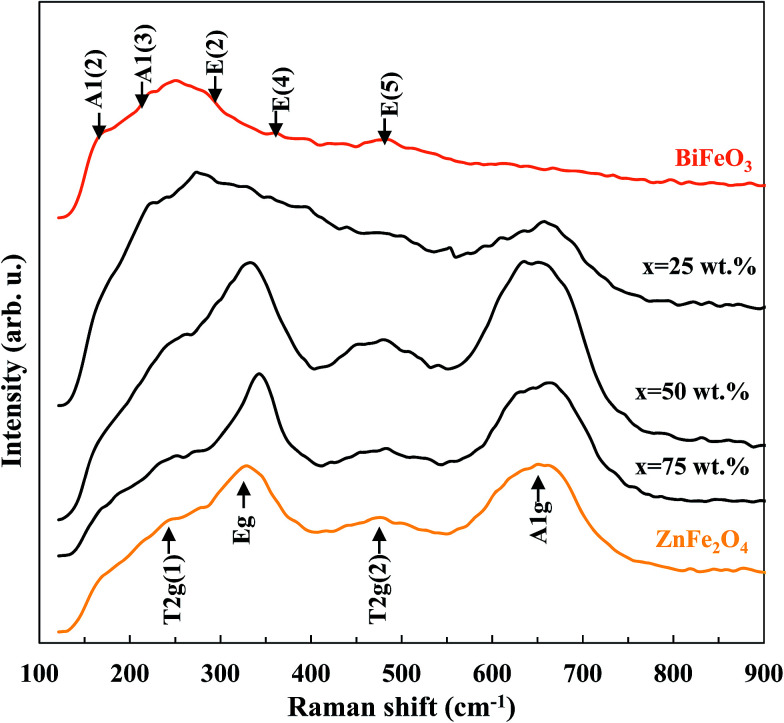
Raman spectra of the BiFeO_3_–*x*ZnFe_2_O_4_ composites.

The Raman spectra of pure BiFeO_3_, pure ZnFe_2_O_4_ and BiFeO_3_–*x*ZnFe_2_O_4_ composites are presented in Fig. 2. In the spectrum of pure BiFeO_3_, the Raman active modes with A_1_ and E symmetry can be summarized using the following irreducible representation *Γ* = 4A_1_ + 9E.^32^ The two peaks at 173 and 220 cm^−1^ are assigned as A1 modes, and the peaks at 286, 361 and 481 cm^−1^ correspond to the E modes. Pure ZnFe_2_O_4_ exhibited four peaks at 246, 327, 471 and 648 cm^−1^, which are assigned as the T_2g_(1), E_g_, T_2g_(2) and A_1g_ modes for a cubic spinel structure, respectively.^33^ The A_1g_ mode of ZnFe_2_O_4_ appears after 25 wt% ZnFe_2_O_4_ was loaded, while the other modes were dominant at higher zinc ferrite contents. Moreover, the purity of the BiFeO_3_–*x*ZnFe_2_O_4_ composites is confirmed by the absence of Raman modes of impurity phases.

**Fig. 3:**
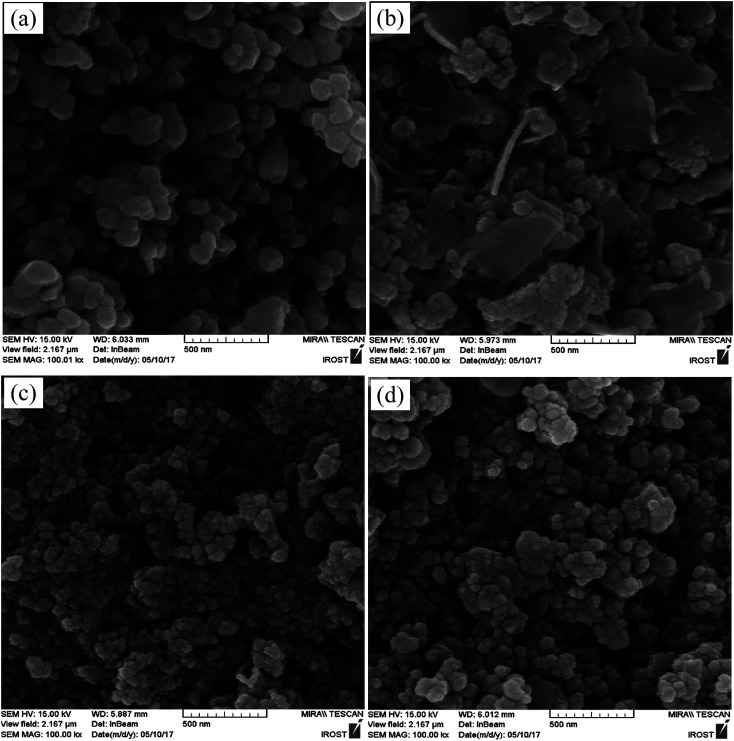
SEM images of (a) pure BiFeO_3_, (b) BiFeO_3_-25 wt% ZnFe_2_O_4_, (c) BiFeO_3_-75 wt% ZnFe_2_O_4_, and (d) pure ZnFe_2_O_4_ powders.

The SEM images of pure BiFeO_3_, BiFeO_3_-25 wt% ZnFe_2_O_4_, BiFeO_3_-75 wt% ZnFe_2_O_4_ and pure ZnFe_2_O_4_ powders are displayed in Fig. 3. The quasi-spherical particles of BiFeO_3_ (210 nm) are larger than the ZnFe_2_O_4_ particles (80 nm). However, the BiFeO_3_-25 wt% ZnFe_2_O_4_ composite is composed of plate-like particles. Furthermore, the average particle size decreases while the particle size distribution becomes rather uniform with an increase in ZnFe_2_O_4_ content.

**Fig. 4:**
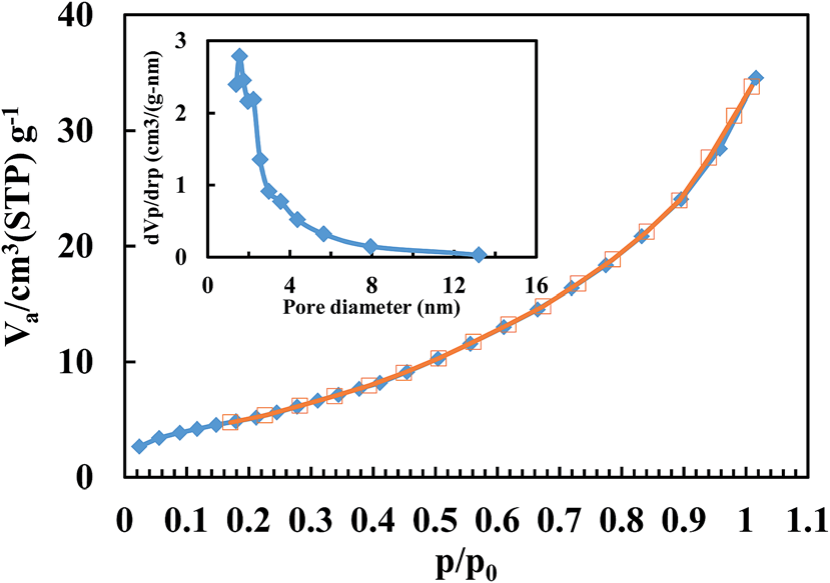
Adsorption (filled symbol)–desorption (open symbol) isotherms of the BiFeO_3_-50 wt% ZnFe_2_O_4_ composite (the inset shows the pore size distribution).

The N_2_ adsorption–desorption isotherms of the BiFeO_3_-50 wt% ZnFe_2_O_4_ composite are shown in Fig. 4. Table 1 also presents the specific surface area (*S*_BET_), equivalent particle size (*D*_BET_) and pore volume. The particle agglomerations show a typical type II isotherm according to the International Union of Pure and Applied Chemistry (IUPAC) classification.^34^ The surface area of pure BiFeO_3_ is 30.56 m^2^ g^−1^ and 13.13 m^2^ g^−1^ for pure ZnFe_2_O_4_. The higher specific surface area of pure BiFeO_3_ is attributed to more gaseous products being formed during its synthesis,^35^ as confirmed by its higher pore volume (0.089 cm^3^ g^−1^). The BJH pore size distribution is also depicted in the inset of Fig. 4. The pore size distribution of the BiFeO_3_-50 wt% ZnFe_2_O_4_ composite powder exhibits a mesopore spreading of about 3–4 nm.

**Table 1:** Dependence of specific surface area, *S*_BET_, pore volume and equivalent particle size, *D*_BET_, on ZnFe_2_O_4_ content (x)

x	*S*_BET_ (m^2^ g^−1^)	Pore volume (cm^3^ g^−1^)	*D*_BET_ (nm)
BiFeO_3_	30.56	0.089	23.6
25 wt%	28.42	0.086	27.9
50 wt%	19.75	0.072	44.8
75 wt%	18.97	0.069	52.6
ZnFe_2_O_4_	13.13	0.053	87.1

**Fig. 5:**
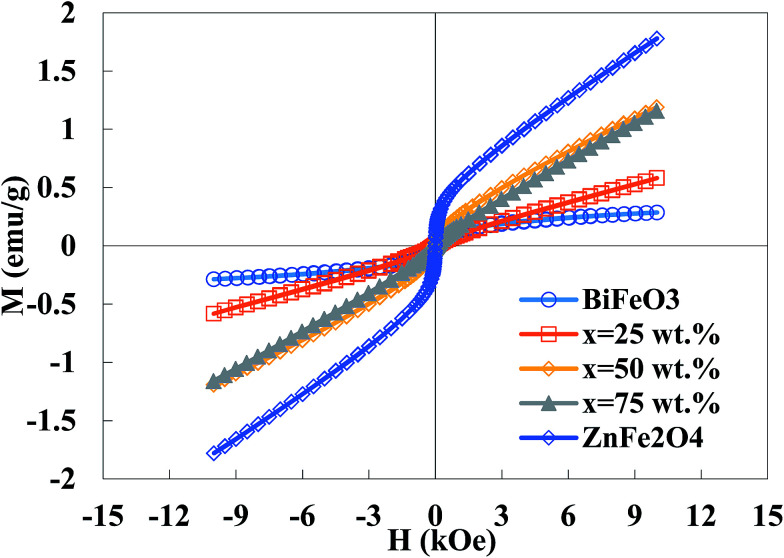
Magnetization curves of the BiFeO_3_–*x*ZnFe_2_O_4_ composites.

Fig. 5 illustrates the magnetization curves of the BiFeO_3_–*x*ZnFe_2_O_4_ composites as well as the pure BiFeO_3_ and ZnFe_2_O_4_ powders. The pure BiFeO_3_ nanoparticles exhibit a ferrimagnetic response with the magnetization of 0.28 emu g^−1^ at 10 kOe. However, the magnetization increases with an increase in zinc ferrite content since pure ZnFe_2_O_4_ has a magnetization of 1.8 emu g^−1^. Bulk BiFeO_3_ is known to show a G-type antiferromagnetic ordering with a linear field-dependence of magnetization, while the BiFeO_3_ nanoparticles exhibit weak ferrimagnetism due to the interruption of the long-range antiferromagnetic order at the particle surface.^36^ The bulk ZnFe_2_O_4_ also has a normal spinel structure with antiferromagnetic behavior, while the ZnFe_2_O_4_ nanoparticles exhibit a partially inverse spinel structure with some magnetic moment at room temperature.^37^ A high surface-to-volume ratio in nanoparticles leads to more uncompensated spins from the surface, inducing an enhancement in magnetization. The BiFeO_3_–*x*ZnFe_2_O_4_ composites show higher saturation magnetization than pure bismuth ferrite as a result of the higher magnetization in the zinc ferrite phase. This ferrimagnetism behavior can be exploited for the magnetic recovery of the photocatalyst after degradation.

**Fig. 6:**
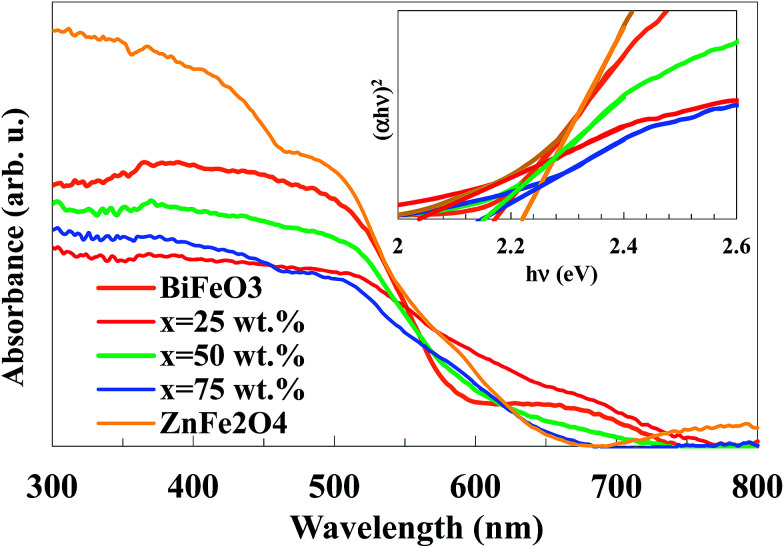
UV-vis absorption spectra of the BiFeO_3_–*x*ZnFe_2_O_4_ composites (the inset shows the Tauc plots).

The optical properties of the BiFeO_3_–*x*ZnFe_2_O_4_ composites, as well as the pure BiFeO_3_ and ZnFe_2_O_4_ powders were investigated *via* UV-vis diffuse reflectance spectroscopy, which are presented in Fig. 6. The absorption spectra show that the samples absorb a considerable amount of visible light. The direct optical band gap, *E*_g_, was determined using the equation (*αhν*)^2^ = *A*(*hν* − *E*_g_), where, *hν* is the photon energy in eV, *α* is the absorption coefficient and *A* is a material constant,^38^ as shown in the inset of Fig. 6. According to the Tauc plots, the band gaps for *x* = 0, 25, 50, 75 and 100 wt% were calculated to be 2.17, 2.03, 2.14, 2.15 and 2.22 eV, respectively. The absorption band of BiFeO_3_ and ZnFe_2_O_4_ is attributed to the electronic transition from the valence band (O 2p orbital) to the conduction band (Fe 3d orbital) (O_2p_^2−^ → Fe_3d_^3+^).^39,40^ Clearly, the band gap of the BiFeO_3_–*x*ZnFe_2_O_4_ photocatalysts gradually decreases with an increase in BiFeO_3_. In other words, by introducing ZnFe_2_O_4_ into BiFeO_3_, the photocatalyst could absorb more visible light for the production of electron–hole pairs, which are favorable for photocatalytic reactions.

**Fig. 7:**
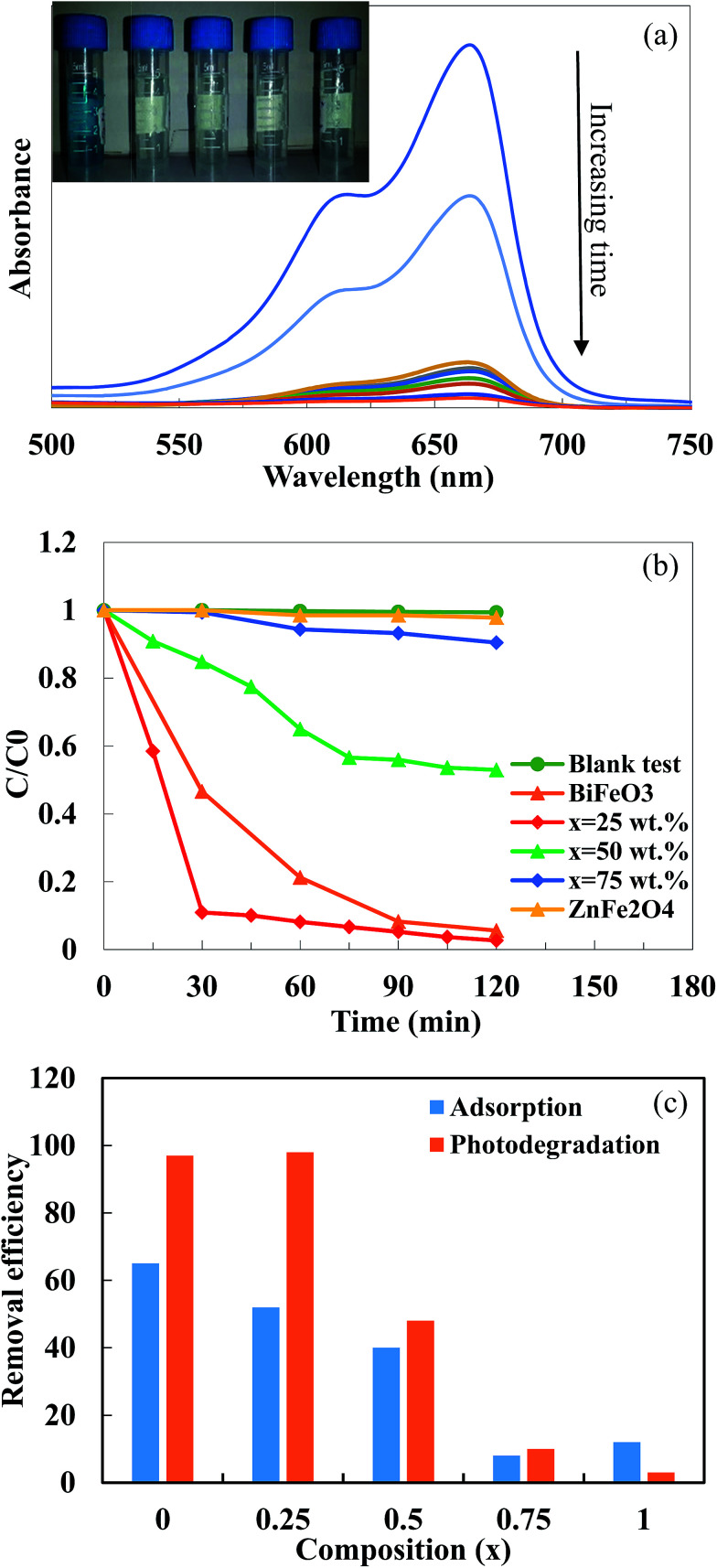
(a) UV-vis spectra of MB solution in the presence of the BiFeO_3_-25 wt% ZnFe_2_O_4_ composite (the inset shows the photodegradation of MB), (b) *C*/*C*_0_*versus* irradiation time for the photodegradation of MB dye under visible light irradiation by the BiFeO_3_–*x*ZnFe_2_O_4_ nanocomposites and (c) the removal efficiency of MB dye by adsorption and photodegradation.

Fig. 7a shows the UV-vis spectra of the MB solution after different irradiation times in the presence of the BiFeO_3_-25 wt% ZnFe_2_O_4_ composite. The main absorption peaks of MB molecules at 664 nm almost completely disappeared after about 120 min, which suggests the excellent photocatalytic activity of the BiFeO_3_-25 wt% ZnFe_2_O_4_ composite. The photodegradation efficiency of MB dye by pure BiFeO_3_, pure ZnFe_2_O_4_ and BiFeO_3_–*x*ZnFe_2_O_4_ composites as a function of irradiation time are summarized in Fig. 7b.

Methylene blue was hardly degraded (∼3%) by pure ZnFe_2_O_4_ which exhibited very limited photolysis of MB under visible light irradiation. The low photocatalytic efficiency of pure ZnFe_2_O_4_ can be attributed to its low valence band potential and poor photoelectric conversion.^7,41^ However, pure BiFeO_3_ can degrade 94.5% of MB after 2 hours of irradiation. The maximum MB photodegradation of ∼97% was observed for the BiFeO_3_-25 wt% ZnFe_2_O_4_ composite after 30 minutes of irradiation. The extraordinary photocatalytic efficiency of the BiFeO_3_-25 wt% ZnFe_2_O_4_ composite may be attributed to the formation of BiFeO_3_–ZnFe_2_O_4_ heterojunctions, which promote the separation of photogenerated electron–hole pairs, thus enhancing the photocatalytic activity. However, the number of effective heterojunctions and thus separation efficiency strongly depend on the content of the two components in the composite.^20–22,42^ For the optimal content of 25 wt% ZnFe_2_O_4_, the most appropriate BiFeO_3_/ZnFe_2_O_4_ heterojunctions might be formed, which benefit the transfer and separation of photogenerated electrons and holes, as can be inferred from the PL spectra.

**Fig. 8:**
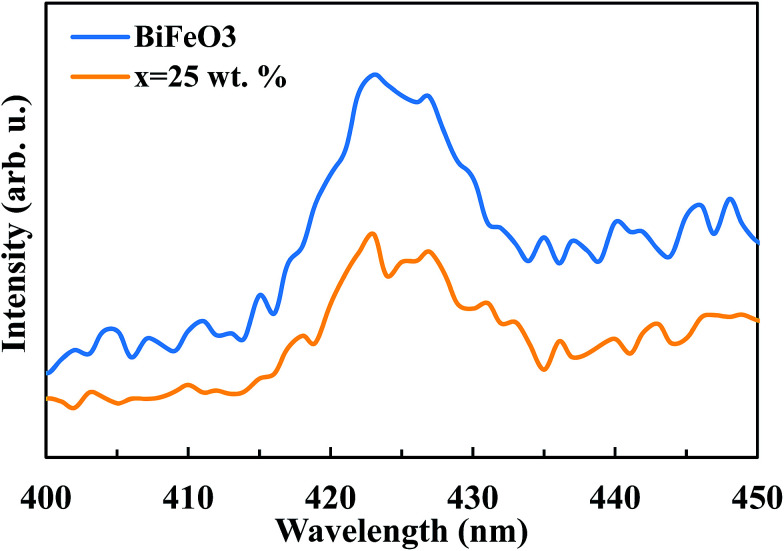
Comparison of the PL spectra of pure BiFeO_3_ and BiFeO_3_-25 wt% ZnFe_2_O_4_ composite.

The suppression of charge recombination in BiFeO_3_ by pairing with ZnFe_2_O_4_ can be confirmed by photoluminescence (PL) emission spectra, as presented in Fig. 8. As is known, the recombination of excited electrons and holes leads to PL emission, where a lower emission intensity indicates a decrease in recombination probability. Fig. 8 shows the PL emission spectra of the pure BiFeO_3_ and BiFeO_3_-25 wt% ZnFe_2_O_4_ photocatalysts at an excitation wavelength of 210 nm. The irradiative recombination process of self-trapped excitations results in an emission band at about 423 nm for pure BiFeO_3_.^43^ Clearly, the PL emission intensity decreases when zinc ferrite was added, which confirms that the coupling of BiFeO_3_ with ZnFe_2_O_4_ results in an enhanced ability to capture photoinduced electrons in comparison with pure BiFeO_3_ and pure ZnFe_2_O_4_. The lower PL emission intensity of the BiFeO_3_-25 wt% ZnFe_2_O_4_ photocatalyst benefits a delay in the recombination rate and, thus, higher photocatalytic activity.^44–47^ In addition to the lower recombination rate of electron–hole pairs in the BiFeO_3_-25 wt% ZnFe_2_O_4_ catalyst, its higher specific surface area can also adsorb more MB dye on the exterior of its particles, as shown in Fig. 7c, hence facilitating the photodegradation of MB dye.

**Fig. 9:**
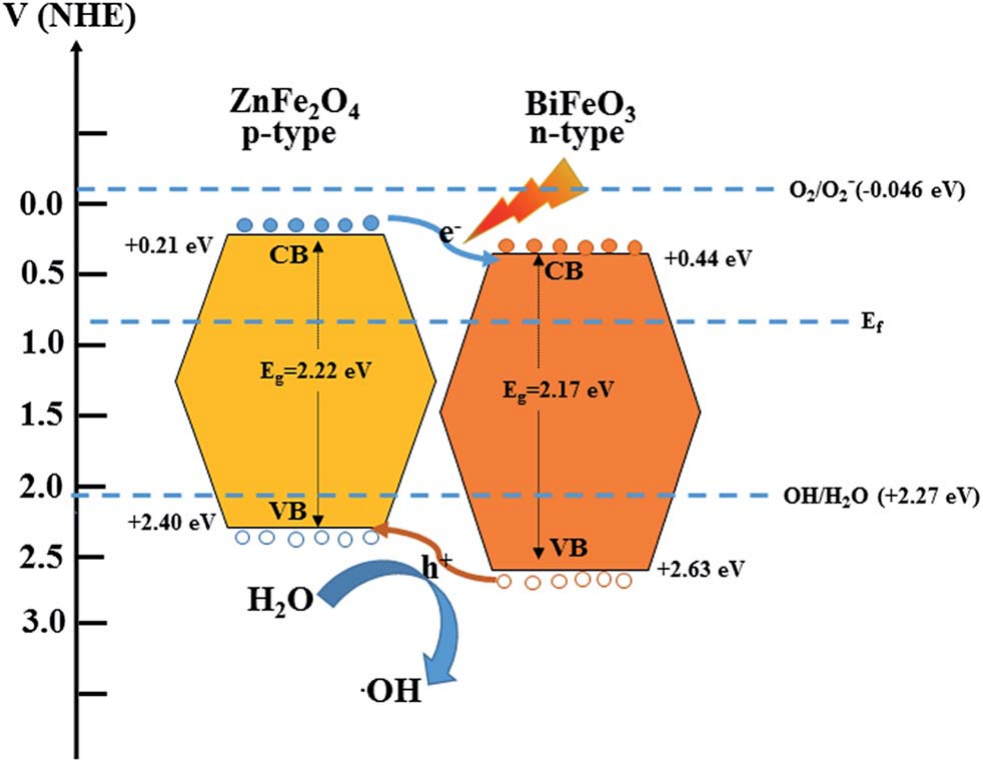
Schematic for electron–hole transport at the interface of the BiFeO_3_–ZnFe_2_O_4_ composite.

Based on the above structural characterizations and visible light photocatalytic tests, a possible mechanism for the photodegradation of MB by the BiFeO_3_/ZnFe_2_O_4_ photocatalyst under visible light irradiation is proposed. Fig. 9 shows the band positions and transfer path of the photogenerated electron–hole pairs between BiFeO_3_ and ZnFe_2_O_4_. The conduction (CB) and valence (VB) band positions of BiFeO_3_ and ZnFe_2_O_4_ at the point of zero charge were obtained from previous reports.^15,48^ According to the general p–n heterojunction formation process,^8^ the entire energy band of BiFeO_3_ increases while that of ZnFe_2_O_4_ decreases to achieve an equilibrium state of the Fermi energy level of BiFeO_3_ and ZnFe_2_O_4_. In this case, the conduction band and valence band of ZnFe_2_O_4_ become higher than that of BiFeO_3_.

Under visible light irradiation, a high energy photon excites an electron from the valence band (VB) to the conduction band (CB) of BiFeO_3_ and ZnFe_2_O_4_. The photoinduced electrons in ZnFe_2_O_4_ can easily transfer to BiFeO_3_, while the holes can transfer to the VB of ZnFe_2_O_4_ from the VB of BiFeO_3_ conveniently with the help of the internal electric field formed at the interface between BiFeO_3_ and ZnFe_2_O_4_.^20^ Therefore, the photogenerated electrons and holes are efficiently separated between BiFeO_3_ and ZnFe_2_O_4_ reducing the electron–hole recombination in the composite photocatalyst, thus improving the photo-oxidation efficiency. The separated holes when moving to the surface of the BiFeO_3_/ZnFe_2_O_4_ composite could react with H_2_O to form hydroxyl radicals, ˙OH, which are powerful oxidative species for the direct oxidation of MB, leading to its decomposition.^49,50^ However, the single electron reduction potential of O_2_ (*E*_0_(O_2_/O_2_^−^) = −0.046 eV) is less negative than the CB potentials, which confirms that the photoinduced electrons on the surfaces of BiFeO_3_/ZnFe_2_O_4_ could not reduce O_2_ to yield O_2_^−^ and could not take part in the photodegradation process.^50,51^ The suitable ZnFe_2_O_4_ content causes good dispersion in the catalyst, which benefits the formation of heterojunctions between the BiFeO_3_ and ZnFe_2_O_4_ particles. Therefore, the high separation of charge carriers leads to the high photocatalytic activity of the BiFeO_3_-25 wt% ZnFe_2_O_4_ photocatalyst.

## 4. Conclusions

A two-pot approach was used for the synthesis of BiFeO_3_/ZnFe_2_O_4_ composites without any impurity species formed between BiFeO_3_ and ZnFe_2_O_4_. The particle size decreased from 210 nm for pure BiFeO_3_ to 80 nm for pure ZnFe_2_O_4_. The pure BiFeO_3_ nanoparticles exhibited a higher specific surface area than the pure ZnFe_2_O_4_ nanoparticles, which may be due to the greater amount of released gaseous products. The magnetization of the BiFeO_3_/ZnFe_2_O_4_ composites increased from 0.28 to 1.8 emu g^−1^ with an increase in the ZnFe_2_O_4_ content. The optical band gaps of composites initially decreased from 2.17 to 2.03 eV and then increased to 2.22 eV as a function of the amount of zinc ferrite. The maximum efficiency (∼97%) for the photodegradation of methylene blue under visible light was exhibited for BiFeO_3_-25 wt% ZnFe_2_O_4_ after 30 minutes irradiation due to the synergic effect between BiFeO_3_ and ZnFe_2_O_4_.

## Conflicts of interest

There are no conflicts to declare.

## Supplementary Material

RA-008-C7RA13380D-s001
