# Supplementary material for: Photocatalytic activity of BiFeO3/ZnFe2O4 nanocomposites under visible light irradiation
Source: RSC Adv. 2018 Feb 13;8(13):6988–95. doi: 10.1039/c7ra13380d (PMC9078354; doi:10.1039/c7ra13380d)
Supplement: RA-008-C7RA13380D-s001 [file RA-008-C7RA13380D-s001.pdf]

Electronic Supplementary Material (ESI) for RSC Advances.
This journal is © The Royal Society of Chemistry 2018

Supporting information for
Photocatalytic activity of $ZnFe_2O_4$/$BiFeO_3$ nanocomposites under visible light irradiation

B. Safizadeh, S.M. Masoudpanah [*], M. Hasheminiasari, A. Ghasemi
School of Metallurgy & Materials Engineering, Iran University of Science and Technology (IUST), Tehran, Iran

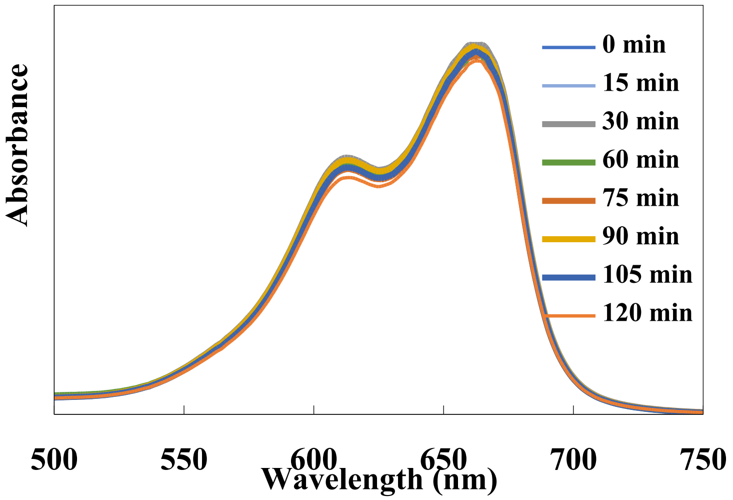

Fig. 1S. UV-Vis spectra of MB after different irradiation times using $BiFeO_3$-25 wt.% $ZnFe_2O_4$ catalyst at pH of 7.

[*] Corresponding author: e-mail: masoodpanah@iust.ac.ir, Phone: +98 21 77240540, Fax:+98 21 77240480

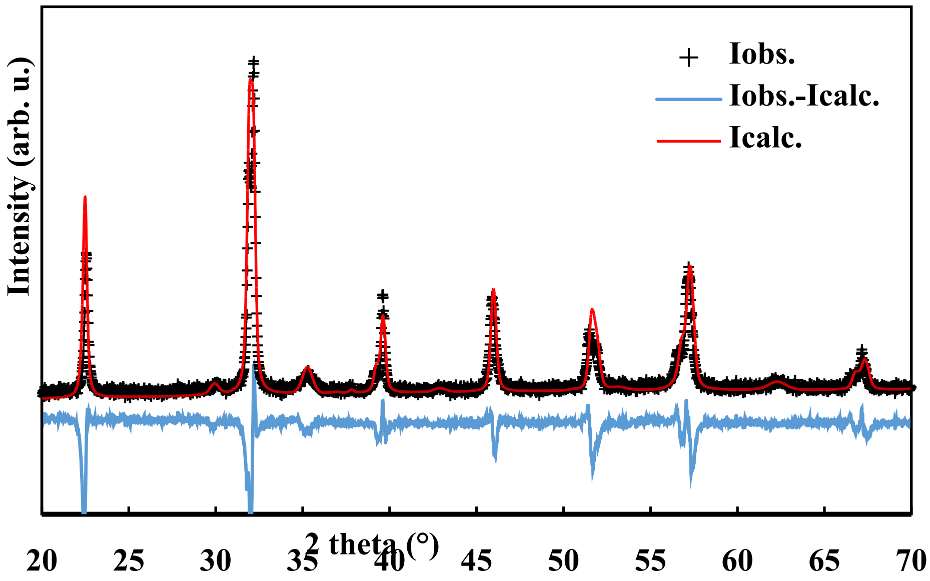

Fig. 2S. XRD pattern and Rietveld results for $BiFeO_3$-25 wt. %$ZnFe_2O_4$ composite. The fitting qualities, Rb, Rwp, and χ^2 are 8.1%, 5.5%, and 1.34, respectively.
